# Identification of a ternary protein-complex as a therapeutic target for K-Ras-dependent colon cancer

**DOI:** 10.18632/oncotarget.2001

**Published:** 2014-05-25

**Authors:** Xiaomei Qi, Congying Xie, Songwang Hou, Gang Li, Ning Yin, Lei Dong, Adrienne Lepp, Marla A. Chesnik, Shama P. Mirza, Aniko Szabo, Susan Tsai, Zainab Basir, Shixiu Wu, Guan Chen

**Affiliations:** ^1^ Department of Pharmacology and Toxicology, Medical College of Wisconsin; ^2^ Department of Radiation Oncology, First Affiliated Hospital, Wenzhou Medical College, Wenzhou, China; ^3^ Department of Biochemistry, Biotechnology & Bioengineering Center, Medical College of Wisconsin, Milwaukee, WI; ^4^ Division of Biostatistics, Medical College of Wisconsin, Milwaukee, WI; ^5^ Department of Surgery, Medical College of Wisconsin, Milwaukee, WI; ^6^ Department of Pathology, Medical College of Wisconsin, Milwaukee, WI; ^7^ Research Services, Zablocki Veterans Affairs Medical Center, Medical College of Wisconsin, Milwaukee, WI; ^8^ The current address at Cancer Center, Hangzhou Cancer Hospital, Zhejiang, China

**Keywords:** p38γ MAPK, Hsp90, K-Ras, ternary complex, therapeutic target, and colon cancer

## Abstract

A cancer phenotype is driven by several proteins and targeting a cluster of functionally interdependent molecules should be more effective for therapeutic intervention. This is specifically important for Ras-dependent cancer, as mutated (MT) Ras is non-druggable and targeting its interaction with effectors may be essential for therapeutic intervention. Here, we report that a protein-complex activated by the Ras effector p38γ MAPK is a novel therapeutic target for K-Ras-dependent colon cancer. Unbiased proteomic screening and immune-precipitation analyses identified p38γ interaction with heat shock protein 90 (Hsp90) and K-Ras in K-Ras MT, but not wild-type (WT), colon cancer cells, indicating a role of this complex in Ras-dependent growth. Further experiments showed that this complex requires p38γ and Hsp90 activity to maintain MT, but not WT, K-Ras protein expression. Additional studies demonstrated that this complex is activated by p38γ-induced Hsp90 phosphorylation at S595, which is important for MT K-Ras stability and for K-Ras dependent growth. Of most important, pharmacologically inhibition of Hsp90 or p38γ activity disrupts the complex, decreases K-Ras expression, and selectively inhibits the growth of K-Ras MT colon cancer in vitro and in vivo. These results demonstrated that the p38γ-activated ternary complex is a novel therapeutic target for K-Ras-dependent colon cancer.

## INTRODUCTION

Mutated (MT) K-Ras is the most established oncogene in human cancer, which occurs in up to 50% of colon cancer [1, 2]. Despite of extensive studies for more than 40 years, MT K-Ras is still non-druggable and there is a complete lack of targeted therapies against K-Ras MT cancer [3, 4]. Moreover, cancers with MT K-Ras are more aggressive [5] and resistant to targeted therapies such as anti-EGFR (epidermal growth factor receptor) [6, 7] and MAPK kinase (MEK) inhibitors [8, 9]. Therefore, there continues to be an urgent need to search for novel targets for therapeutic development against K-Ras MT cancers. Since an activated onco-protein requires interaction with crucial partners for oncogenic activity [10-12], targeting a complex of functionally interdependent molecules may be a strategy for treatment of K-Ras dependent cancer.

Multiple pathway activities contribute to Ras oncogenesis, including MAPK (mitogene-activated protein kinase), PI3K (phosphatidylinosital 3-kinase)/Akt, and PKC (protein kinase C) cascades [1, 13]. Exact mechanisms by which these kinases regulate Ras oncogenisis, however, have not been demonstrated. p38 MAPKs consist of four family proteins (α, β, γ, and δ). In contrast to the tumor suppressor role of p38α [14-16], p38γ is required for Ras oncogene activity [17-20]. In a manner different than the classical MAPK activation by phosphorylation [21], p38γ expression and phosphorylation are both increased in K-Ras MT human colon cancer [22]. Recent studies further showed that p38γ is overexpressed in several types of human cancers such as colon cancer [20, 23], breast cancer [24-26], liver cancer [27], and gliomas [28]. Furthermore, we recently showed that p38γ binds and phosphorylates several proteins important for cancer growth and progression including protein tyrosine phosphatase H1 (PTPH1) [22], DNA topoisomerase IIα [29], and estrogen receptor [24]. These results indicate that p38γ may promote the malignant growth through interacting and phosphorylating its substrates. However, the exact mechanism by which activated p38γ promotes the K-Ras dependent colon cancer growth remains unknown.

Heat shock protein 90 (Hsp90) is a highly conserved molecular chaperone that stabilizes and thereby activates more than 200 client proteins [30]. Hsp90 uses the energy generated by ATP binding/hydrolysis to fold its client protein in a proper structure through cooperation with its co-chaperones [31]. In doing so cancer cells protect an array of mutated and/or overexpressed oncoproteins from misfolding and degradation thereby maintaining the constitutively proliferative signaling [30, 31]. Indeed, many of Hsp90 clients are proliferative [32-34] and/or transforming [34, 35] kinases or activated oncoproteins. Despite the central role of K-Ras in human cancer, a direct connection between K-Ras mutations and Hsp90 signaling has not been reported.

Hsp90 proteins from tumor cells are about 100-fold more potent in binding to its inhibitor 17-alllylaminogeldanamycin (17-AAG) than those from normal cells [36], indicating a potential of Hsp90 inhibitors in cancer therapy [37, 38]. However, Hsp90 inhibitors are frequently associated with toxicities and poor selectivity, likely as a result of abundant Hsp90 client proteins in cancer cells [12, 38]. Recent studies showed that Hsp90 phosphorylation can increase its specificity to a certain client without affecting others [39]. Here, we tested the hypothesis that the Ras effector p38γ may drive K-Ras dependent colon cancer growth by activating an oncogenic complex through Hsp90. Our results showed that p38γ specifically forms a complex with Hsp90 and K-Ras in K-Ras MT colon cancer cells through activating Hsp90 by phosphorylation. Disrupting of this ternary-complex by targeting Hsp90 or p38γ decreases MT K-Ras expression and selectively inhibits K-Ras dependent colon cancer growth *in vitro* and/or *in vivo*. These results demonstrated that a ternary protein-complex activated by the Ras effector p38γ is a therapeutic target for K-Ras dependent colon cancer.

## Results

### p38γ overexpression in colon cancer correlates with a shortened survival and increased metastasis, and p38γ forms a complex with Hsp90 and K-Ras in K-Ras MT but not WT colon cancer cells

**Figure 1:**
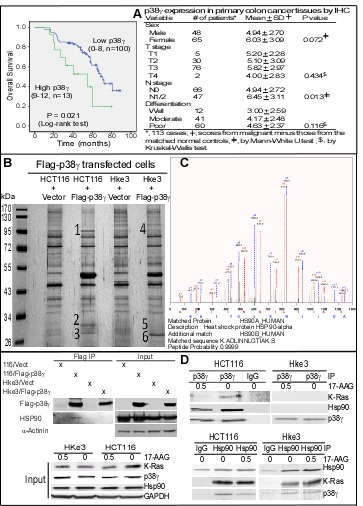
p38γ overexpression in CRC specimens correlates with shortened patient survival and p38γ specifically forms a ternary complex with Hsp90 and K-Ras in K-Ras MT colon cancer cells (A) Colon cancer specimens were stained for p38γ expression by IHC [20] and Kaplan-Meier plot of the overall survival according to p38γ levels was shown (left), and associated clinical parameters were given at left. (B, C) Flag immune-precipitates were separated by SDS-PAGE and samples from the numbered region (1-6) were digested, which were then subjected to proteomic analysis as described [22]. MS/MS spectra of heat shock protein 90α peptide HLEINPDHSIIETLR and 90β peptide ADLINNLGTIAK with representative peak matches for b and y ions {detected from the sample 1 but not sample 4 of B}. Additional proteomic data were given in Supplementary Figure S1B/C). (D) Western blotting (WB) analysis of Flag precipitates revealed the p38γ interaction with Hsp90 in HCT116 but not in Hke-3 cells (top left). Endogenous p38γ and Hsp90 proteins were also isolated after treatment with 17-AAG or DMSO for their interactions with K-Ras (top right and bottom right) with a portion of whole cell lysates analyzed by direct WB as an input (bottom, left).

Our previous studies have shown that p38γ is overexpressed in about 80% of primary colon cancers [20]. Here, we wanted further to examine if overexpressed p38γ has a prognostic activity in clinical colon cancer. In this regard, a group of specimens were first analyzed by immunohistochemistry (IHC) for p38γ expression [20, 23]. To examine the relationship between p38γ expression and patients' survival, the overall survival time was plotted according to p38γ expression levels [20, 23]. Results in Figure 1A (left) (Supporting information, Figure 1A) showed that increased p38γ expression correlates significantly with a shortened survival, with the 5-year overall survival rate of 19.8% in the high p38γ group versus 61.8% in the low p38γ group (P < 0.05). Moreover, levels of p38γexpression are also significantly increased in lymph-node positive colon cancer (Figure 1A, right). These results together indicate that p38γ overexpression is a poor prognosis marker for clinical colon cancer. Because elevated p38γ is required for K-Ras transformation [17, 20], we wanted next to determine if up-regulated p38γ distinctively regulates the growth of colon cancer cells with and without K-Ras mutation through interacting with a different set of proteins.

To explore this possibility, we stably expressed Flag-p38γ in the isogenic cell-line pair HCT116 and Hke3. HCT116 are colon cancer cells with a wild-type (WT) K-Ras and a mutant (MT) K-Ras (K-Ras^D13^) allele. Somatic deletion of the K-Ras^D13^ allele generated Hke3 cells with a reverted oncogenic phenotype *in vitro* and *in vivo* [42]. Flag antibody-isolated precipitates were then subjected to proteomic analysis to screen for MT K-Ras dependent p38γ binding partners [20]. Results in Figure 1B/C (Supporting information, Figure 1B/C) showed that the precipitates from HCT116, but not from Hke3 cells, contained Hsp90, as revealed by detection of three peptides from Hsp90α protein in which two of them also match to its family member Hsp90β sequence. These results were further confirmed by Western Blot (WB) analysis of Flag precipitates using an antibody reactive both with Hsp90α and Hsp90β (defined as Hsp90) (Figure 1D, top left), indicating a MT K-Ras dependent Hsp90-binding activity of p38γ. Analysis of endogenous p38γ precipitates (Figure 1D, right) further showed that p38γ binds Hsp90 and K-Ras in HCT116 but neither in its MT K-Ras disrupted Hke3 line [42, 43]. Although Hsp90 precipitates contain p38γ and K-Ras in both cell lines, an inhibition of Hsp90 activity with 17-AAG only decreases levels of the associated K-Ras and p38γ in 116 cells (Fig*ure 1D*, bottom right). Significantly, incubation with 17-A significantly down-regulates K-Ras protein expression in whole cell lysates (input) in HCT116, but not Hke3, cells (Figure 1D, bottom left). These results revealed that p38γ forms a 17-AAG sensitive ternary complex with Hsp90 and K-Ras only in K-Ras MT cells, indicating its role in transduction of MT K-Ras signaling to Hsp90.

### MT K-Ras is an Hsp90 client protein and the Hsp90 inhibitor 17-AAG selectively suppresses K-Ras-dependent colon cancer growth in *in vitro* and/or *in vivo*

**Figure 2:**
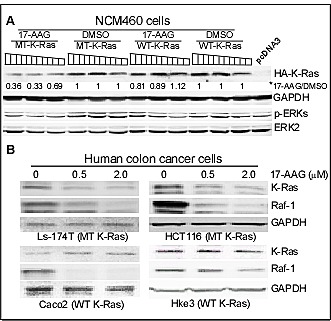
K-Ras is converted to an Hsp90 client protein upon mutation (A) NCM460 cells were transiently expressed with increasing concentrations (0.5, 1.0, and 1.5μg DNA) of WT and MT (G12V) K-Ras constructs for 24h, followed by incubation with 0.1 μM of 17-AAG or DMSO for additional 24 h, which were then subjected to WB analysis. *, the band intensity was quantitated by the NIH software, which was normalized with GAPDH, and K-Ras protein level in 17-AAG-treated group was expressed as relative to that treated with DMSO. A separate experiment showed similar results. (B) Colon cancer cells with indicated K-Ras phenotype were incubated with indicated concentration of 17-AAG for 24 h, which were then analyzed for protein expression by WB. Similar results were obtained in a separate experiment.

Hsp90 plays a central role in protection of mutated and activated onco-proteins from degradation [30]. Its role in protecting MT K-Ras protein, however, has never been demonstrated. The presence of K-Ras protein in the p38γ/Hsp90 precipitates and its down-regulation by 17-AAG in HCT116 (but not Hke3) cells suggest that K-Ras may be converted into an Hsp90 client protein upon mutation through p38γ actions. To demonstrate if K-Ras mutation alone is sufficient to trigger this conversion, increasing amounts of WT and MT (G12V) K-Ras in pcDNA3 vector were transiently expressed in normal human colon epithelial NCM 460 cells. Cells were then incubated with and without 17-AAG, and analyzed for protein expression by WB. Results in Figure 2A showed that levels of expressed HA-tagged MT K-Ras protein are significantly down-regulated by 17-AAG, whereas those of WT K-Ras are only minimally affected, indicating that K-Ras is converted into an Hsp90 client protein upon mutation. To further demonstrate if endogenous MT K-Ras is an Hsp90 client, colon cancer cells harboring WT or MT K-Ras [20] were treated with 17-AAG and effects on the Ras expression were analyzed by WB. Results in Figure 2B showed that incubation with 17-AAG only down-regulates K-Ras in K-Ras MT cells but decreases Raf-1 {an established Hsp90 client [32]} in all cell lines. These results, for the first time, demonstrated that both transfected and endogenous MT K-Ras is an Hsp90 client protein.

**Figure 3:**
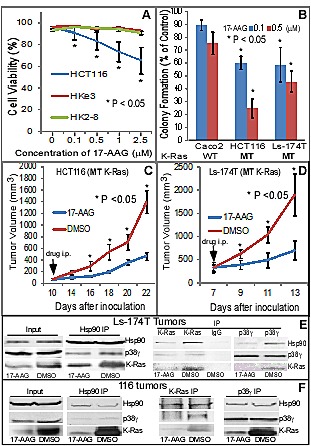
Hsp90 is a therapeutic target for K-Ras MT colon cancer (A) HCT116 and its MT K-Ras disrupted sub-lines were incubated with 17-AAG for 24 h and assessed for cell viability using trypan blue staining [22]. Results are means of three experiments (+ SD, * vs. either Hke3 or HK2-8 cell line). (B) Indicated cells were cultured in the presence and absence of 17-AAG for about 2 weeks and colony formed was manually counted. Results are expressed as % of solvent treated control (mean ± SD, n = 3, * vs. Caco2 cells). (C, D) Indicated human colon cancer cells (2 × 10^6^ in 0.1 ml of PBS) were s.c. injected into nude mice on both sides of front flank and 17-AAG (80 mg/kg) or solvent (DMSO) solution (in 50 μl) was i.p. administrated daily for 14 days for HCT116 tumors and for 6 days for Ls-174T xenograft. Changes in tumor volume and body weight were monitored every other day [20]. Results in all groups are means of 4-5 tumors (± SEM, n = 4-5 mice, * vs. DMSO). (E, F) The same amounts of lysate proteins from DMSO or 17-AAG treated tumors were immune-precipitated with indicated antibodies and precipitates were then analyzed by WB for their complex formation, with a portion of whole lysates analyzed by direct WB as an input.

To investigate if Hsp90 may act as a therapeutic target for K-Ras MT colon cancer, HCT116 and its two MT K-Ras disrupted sub-lines [42, 44] were incubated with 17-AAG and effects on cell-death were determined. Results in Figure 3A showed a 17-AAG dose-dependent decrease in cell viability in HCT116, but not in Hke3 and HK2-8, cells. Moreover, a re-expression of the MT K-Ras (G12V) in Hke3 cells increases p38γ expression and enhances the sensitivity to 17-AAG (Supporting information, Figure S2A). These results indicate a sufficient role of MT K-Ras in inducing p38γ expression and in increasing the 17-AAG induced growth-inhibition in human colon cancer cells. An increased toxicity to 17-AAG was further demonstrated by a long-term colony formation assay in two MT K-Ras colon cancer cell lines as compared with WT K-Ras Caco2 cells (Figure 3B). To further demonstrate the therapeutic activity of 17-AAG in K-Ras MT colon cancer *in vivo*, HCT116 and Ls-174T cells were injected into nude mice and the inhibitor was systemically administrated after tumors became palpable. Results in Figure 3C/D (and Supporting information, Figure S2B) showed that 17-AAG significantly inhibits the growth of both xenografts without significant effects on mouse body weight (data not shown). Furthermore, analyses of immune-precipitates from tumor lysates by an antibody against p38γ, Hsp90 or K-Ras revealed that there is a ternary-complex formation, which is disrupted by the 17-AAG treatment in both tumors, leading to a down-regulation of K-Ras expression (Figure 3E/F). These results together demonstrated that MT K-Ras is an Hsp90 client protein and Hsp90 may be a novel therapeutic target for K-Ras-dependent colon cancer through a complex formation with p38γ and K-Ras.

### p38γ activates Hsp90 by inducing its phosphorylation at Ser595, which is important both for K-Ras protein expression in K-Ras MT cells and for K-Ras dependent colon cancer growth

**Figure 4:**
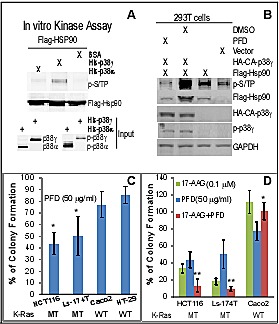
p38γ phosphorylates Hsp90, and the p38γ pharmacological inhibitor pirfenidone (PFD) blocks Hsp90 phosphorylation, more effectively inhibits the K-Ras dependent growth *in vitro*, and selectively synergizes with 17-AAG to inhibit the colony formation of K-Ras MT colon cancer cells (A) Flag-Hsp90 proteins isolated from 293T cells were incubated with bacterially expressed His-p38γ or His-p38α, and kinase activity assays were performed [24]. Phosphorylated Hsp90 was detected by WB using a specific phospho-MAPK substrate (p-S/TP) antibody (left, top) and the same amounts of His-p38α and His-p38γ proteins were analyzed by WB as input controls (left, bottom). (B) Indicated constructs were transfected into 293T cells for 48 h, with 20 μg/ml of PFD or solvent added for the last 24 h, and cells were then analyzed for protein expression and phosphorylation (right). (C, D) Colon cancer cells were incubated with indicated concentrations of PFD and/or 17-AAG for about 2 weeks. Colonies formed were manually counted and presented as % of solvent treatment. Results are mean of 3-4 experiments (± SD, * P < 0.05 vs. either WT K-Ras line for C; ** P < 0.05 vs. either inhibitor, * P > 0.05 vs. either inhibitor for D).

Hsp90 activity is regulated by phosphorylation [45]. Because levels of p-p38γ protein expression are associated with those of its substrate protein tyrosine phosphatase H1 phosphorylation (p-PTPH1) in K-Ras MT, but not WT, colon cancer cells [22], we next examined if p38γ may phosphorylate Hsp90 thereby increasing its chaperone activity toward MT K-Ras. In this case, Flag-tagged human Hsp90α [39] was transiently expressed in 293T cells and Flag precipitates were incubated with His-tagged p38γ *in vitro* by including His-p38α for comparison [22]. Phosphorylated Hsp90 proteins were separated in SDS-PAGE and detected with a specific phospho-MAPK substrate antibody (p-S/TP) [46]. Results in Figure 4A showed that there is an increased phosphorylated band at about 90 kDa after incubation His-p38γ, whereas His-p38α addition has little effect, indicating that Hsp90 is specifically phosphorylated by p38γ *in vitro*. To demonstrate if p38γ phosphorylates Hsp90 *in vivo*, Flag-Hsp90α was co-transfected with a constitutively active (CA) p38γ (the MKK6-p38γ fusion construct) [19] in 293T cells. Cells were then incubated with or without a specific p38γ pharmacological inhibitor pirfenidone (PFD) [47] and analyzed for Hsp90 phosphorylation. Results in Figure 4B showed that levels of phosphorylated Hsp90 (p-Hsp90) are significantly increased by the CA p38γ transfection, which, however, is completely blocked by incubation with PFD. The increase and the decrease in p-Hsp90 expression by the p38γ activation and inhibition are also associated with up- and down-regulation of the total Flag-Hsp90 protein expression (Figure 4B), indicating that p38γ may increase Hsp90 protein expression by phosphorylation. The p-Hsp90 band in the absence of the transfected Flag-Hsp90 (Figure 4B, lane 1 from right) may result from the endogenous Hsp90. These results together demonstrate that p38γ phosphorylates Hsp90 *in vitro* and *in vivo*.

The sequence analysis showed that human Hsp90α contains a single S/TP site (Ser595), a typical MAPK phosphorylation motif [21]. To explore if p38γphosphorylates Hsp90 at this site, a mutated Hsp90/S595A was generated by site-directed mutagenesis using the PCR-based technique [24]. The WT and MT Hsp90 constructs were then co-transfected with Flag-p38γ in 293T cells, which were examined for p-Hsp90 expression [46]. Results (Supporting information, Figure 3A, left) showed that p38γ only phosphorylates WT Hsp90 but not Hsp90/S595A mutant even though two proteins are expressed at a similar level, indicating that p38γ phosphorylates Hsp90 at S595. The lack of an elevation of the exogenously expressed Hsp90 after its phosphorylation may be due to low levels of Hsp90/S595 phosphorylation as a result of Flag-p38γ (instead of its CA form) used in the co-transfection (Figure 4B vs. Supporting information, Figure S3A). Importantly, stable expression of Hsp90/S595A in two K-Ras MT colon cancer cell lines decreases endogenous K-Ras expression as compared to the WT Hsp90 transfection and confers a resistance to 17-AAG-induced inhibition of colony formation and cell growth (Supporting information, Figure S3A-C). These results together demonstrated a critical role of the p38γ-induced Hsp90/S595 phosphorylation in MT K-Ras protein expression and in K-Ras-dependent colon cancer growth.

### The p38γ inhibitor pirfenidone (PFD) preferentially inhibits the colony-formation in K-Ras MT colon cancer cells and synergizes with 17-AAG to specifically inhibit the K-Ras dependent growth *in vitro*

Previous studies showed that PFD, an anti-inflammatory agent [48], specifically inhibits p38γ (but not p38α or p38β) kinase activity *in vitro* [47]. Consistent with this report, we recently showed that PFD efficiently blocks the p38γ phosphorylation of PTPH1 at S459 in colon cancer cells [22]. Because levels of phosphorylated p38γ protein expression are up-regulated in K-Ras MT cells [20] and PFD efficiently inhibits the p38γ-induced Hsp90 phosphorylation (Figure 4B), we next examined if PFD more effectively inhibits the K-Ras dependent growth. Of great interest, results in Figure 4C showed a greater inhibition of colony formation by PFD in two MT K-Ras cell lines than in their WT K-Ras counterparts. The growth-inhibitory effects of PFD in K-Ras MT cells are similar to those observed by p38γ knockdown using shRNA [20]. Because p38γ phosphorylates and activates Hsp90, and Hsp90 stabilizes MT (but not WT) K-Ras protein (Figure 2/4A/4B), we next explored if PFD may have a synergistic growth-inhibitory activity with 17-AAG depending on K-Ras mutation through their respective inhibition of p38γ and Hsp90 activity in the ternary complex. Of great interest, results in Figure 4D (and Supporting information, Figure S4) indeed showed that a combination of PFD with 17-AAG has a greater growth-inhibitory effect than either alone in K-Ras MT, but not WT, colon cancer cells. These results together indicate that a combined targeting Hsp90 and its activator p38γ may be a more effective therapeutic strategy against K-Ras dependent colon cancer.

### p38γ activity is required for MT, but not WT, K-Ras protein expression through a complex formation with Hsp90

We have shown that p38γ phosphorylates Hsp90 (Figure 4A/B) and expression of the p38γ non-phosphorable Hsp90/S595A construct or application of the Hsp90 inhibitor 17-AAG decreases MT-K-Ras protein expression (Figure 2B and Supporting information, Figure S3A, right). These results indicate that p38γ may increase MT, but not WT, K-Ras protein expression by a phosphorylation-dependent mechanism. To demonstrate this possibility, 293T cells were transiently transfected with Flag-p38γ together with and without HA-tagged WT and MT K-Ras, and resultant effects on the ectopic Ras protein expression were examined by WB. Results (Supporting information, Figure 5A) showed that p38γ significantly increases the MT K-Ras expression, whereas it only minimally impacts the WT K-Ras level. Moreover, analysis of HA immune-precipitates showed that only MT, but not WT, K-Ras binds transfected Flag-p38γ, but not its AGF mutant (Supporting information, Figure 5B). These results together indicate that p38γ increases MT K-Ras expression through a complex formation by a phosphorylation-dependent mechanism.

**Figure 5:**
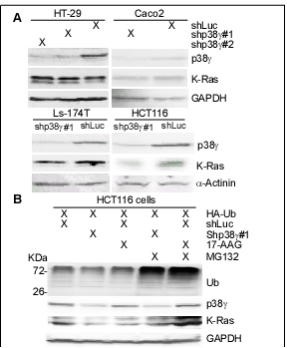
p38γ depletion selectively depletes K-Ras protein in K-Ras MT colon cancer cells (A) Colon cancer cells were stably depleted of endogenous p38γ protein by lentiviral mediated shRNA delivery [20] and analyzed for protein expression by WB. Similar results were obtained in a separate experiment. (B) HCT116 cells with and without p38γdepletion were transiently transfected with HA-UB for 48 h with and without incubation with 17-AAG (0.1 μM) for the last 24 h, followed by the last 6-h incubation with and without MG132 (100 μM). Cell lysates were collected for WB analyses. (C) Colon cancer cells were incubated with 100 μg/ml of PFD or solvent DMSO for 24 h, and then analyzed for protein expression by WB. Similar results were obtained in a separate experiment.

To investigate if endogenous p38γ is also required for MT K-Ras protein expression, colon cancer cells were depleted of p38γ by retroviral mediated shRNA expression [20], which were then examined for K-Ras protein expression by WB. Results in Figure 5A showed that p38γ depletion decreases K-Ras expression only in cells expressing a MT K-Ras. Moreover, the down-regulated K-Ras by 17-AAG and p38γ shRNA was both reversed by treatment with MG132, a proteasome inhibitor (Figure 5B), indicating that p38γ and Hsp90 act by the same proteasome-dependent mechanism to protect MT K-Ras from degradation. In addition, incubation with PFD or 17-AAG decreases the ectopically expressed MT K-Ras protein expression (Supporting information, Figure S5C). These results together indicate that p38γ and Hsp90 both protect MT K-Ras protein from proteasome-dependent degradation.

### PFD inhibits the xenograft-growth of K-Ras dependent colon cancers in nude mice, disrupts the p38γ/Hsp90/K-Ras complex, and decreases MT K-Ras protein expression in tumor tissues

**Figure 6:**
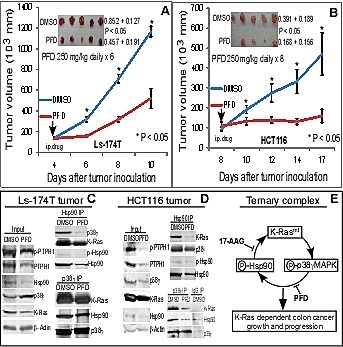
PFD inhibits K-Ras-dependent colon cancer growth in mice, disrupts the p38γ/Hsp90/K-Ras complex, and depletes K-Ras protein in tumor tissues (A, B) Tumor cells were s.c. injected as described above and daily therapy with PFD or DMSO control was i.p. administrated into mice when tumor becomes palpable. Tumor volume was measured every other day and results shown are mean of 4-5 tumors (± SEM). Tumors were also weighted at the end of experiments (inserts, mean ± SD, * vs. PFD). (C, D) PFD disrupts the ternary protein-complex and decreases K-Ras protein expression in tumor tissues. The same amount of lysate proteins from PFD or DMSO treated tumor were subjected to IP with indicated antibodies and precipitates were analyzed for complex formation by WB with a portion of total lysates as an input control. (E) An experimental model elucidates that K-Ras mutation stimulates p38γ expression/phosphorylation leading to Hsp90 phosphorylation resulting in the K-Ras stabilization by a ternary complex. An inhibition of p38γ activity by PFD and/or Hsp90 by 17-AAG will attenuate the complex formation, decrease the K-Ras expression, and suppress the K-Ras dependent growth and progression.

PFD is a non-toxic and orally active anti-fibrotic agent, and is currently in phase III clinical trials for treatment of lung fibrosis [49-52]. Of great interest, we showed that PFD inhibits p38γ phosphorylation of Hsp90 (Figure 4B), decreases MT K-Ras protein expression (Supporting information, Figure S5C), and selectively suppresses the K-Ras dependent growth in cell culture (Figure 4C/D). We wanted next to determine if PFD has a therapeutic activity against K-Ras MT colon cancer in mice and whether it can target the p38γ/Hsp90/K-Ras complex in tumor tissues. In this regard, HCT116 and Ls-174T cells were injected into nude mice, which then received the daily i.p. therapy with PFD or solvent DMSO. Results in Figure 6B showed that the treatment with PFD almost completely blocked the HCT116 xenograft growth. Despite the rapid growth of the Ls-174 tumor likely as a result of its inoculation in Matrigel, the PFD treatment also led to a significant growth inhibition at every time point after the drug administration (Figure 6A). In both cases, there were no drug-associated toxicities. These results demonstrated a strong growth inhibitory activity of the non-toxic p38γ inhibitor PFD in two K-Ras MT colon cancer xenografts.

To demonstrate if PFD also regulates the complex formation, lysates were prepared from tumors and analyzed by Hsp90/p38γ IP/WB. Results in Figure 6C/D showed that the PFD treatment decreases the Hsp90 binding activity both with p38γ and K-Ras, and similar results were also obtained when p38γ immune-precipitates were analyzed. Levels of total and phosphorylated PTPH1 protein expression were decreased in total tumor lysates by the PFD treatment in both tumors (Figure 6C/D), indicating that the p38γ activity is inhibited leading to a decreased PTPH1 phosphorylation and expression [22]. Different than the 17-AAG therapy (Figure 3), the PFD treatment also decreases Hsp90 expression in total tumor lysates as observed in 293T cells (Figure 4B), indicating that it may inhibit the Hsp90 phosphorylation in tumors, which was confirmed by WB analysis of Hsp90 precipitates (Figure 6C/D). Most importantly, levels of K-Ras protein expression in the Hsp90 and p38γ complexes as well as in whole tumor lysates were all significantly down-regulated by the PFD treatment in both tumors. These results are similar to those obtained after the treatment with the Hsp90 inhibitor 17-AAG (Figure 3E/F). Therefore, targeting the p38γ/Hsp90/K-Ras ternary complex by a p38γ or an Hsp90 inhibitor led to a similar growth-inhibition in K-Ras MT colon cancer by mechanisms converged at down-regulating MT K-Ras protein expression.

## Discussion

K-Ras mutation is the most frequently oncogenic event in human cancer. However, thus far there have been no therapeutic strategies effective against K-Ras dependent malignancies [2]. Here, our studies provide several pieces of evidence that together indicate that a p38γ-activated ternary-complex with Hsp90 and K-Ras may be a novel therapeutic target for K-Ras dependent colon cancer. First, unbiased proteomic screening identified Hsp90 as a MT K-Ras dependent binding partner of p38γ (Figure 1B/C), an established Ras effector that is overexpressed in primary colon cancer [20] and has a poor prognostic activity (Figure 1A). The complex was further shown to contain K-Ras protein and a pharmacological inhibitor of p38γ or Hsp90 selectively down-regulates K-Ras expression in K-Ras MT cells (Figure 4C/2/5). Additional experiments showed that p38γ activates Hsp90 by direct phosphorylation and both p38γ and Hsp90 maintain K-Ras expression in K-Ras MT cells via inhibition of proteasome-dependent degradation (Figure 4/5). Moreover, the Hsp90 inhibitor 17-AAG and the p38γ inhibitor PFD are both more effective in inhibition of K-Ras MT colon cancer growth *in vitro* and their combination only has a synergistic growth-inhibitory activity in colon cancer cells harboring a MT K-Ras (Figure 3/4). Most significantly, both 17-AAG and PFD significantly inhibit K-Ras dependent growth in mice and attenuate the p38γ/Hsp90 binding with K-Ras leading to K-Ras protein depletion in K-Ras MT tumor tissues (Figure 3/6). These results together demonstrate that a ternary complex activated by the Ras effector p38γ is a therapeutic target for K-Ras MT colon cancer (Figure 6E).

Targeting several proteins at the same time by different means for therapeutic intervention has been previously explored [37, 53]. Our results, however, for the first time, demonstrated that an interdependent protein-complex initiated by MT K-Ras, activated by its effector p38γ, and coordinated and enhanced by Hsp90 acts as a functional oncogene and thus a valid therapeutic target for K-Ras mutated colon cancer (Figure 6E). This conclusion is based on the fact that immune-precipitates of either protein from the ternary complex contained the other two (Figure 1D/3E/3F/6C/6D). In this complex, K-Ras activating mutation may act as a driver, which leads to p38γ overexpression [17, 20] (Supporting information, Figure 2A) [22]. Activated p38γin the complex may act as an activator to facilitate MT K-Ras to become an Hsp90 client protein by activating Hsp90 through phosphorylation thereby sustaining MT K-Ras expression and maintaining K-Ras constitutively proliferative signaling through a complex formation (Figure 3/4). This is because 1) p38γ overexpression increases and its depletion decreases MT, but not WT, K-Ras expression (Figure 5A and Supporting information, Figure S5B); 2) the stimulating effect of p38γ on MT K-Ras protein expression couples with their binding activity with Hsp90 by a mechanism depending on p38γ phosphorylation and K-Ras mutation (Figure 1D and Supporting information, Figure S5A/B); 3) p38γ phosphorylates Hsp90 *in vitro* and *in vivo* and an Hsp90 mutant that cannot be phosphorylated by p38γ also inhibits MT K-Ras expression and attenuates the K-Ras dependent growth (Figure 4A/B and Supporting information, Figure S3); 4) the p38γ inhibitor PFD blocks the Hsp90 phosphorylation (Figure 4B), disrupts the K-Ras/p38γ/Hsp90 complex, depletes MT K-Ras protein expression (Figure 6C/D), and preferentially inhibits K-Ras MT colon cancer cell growth (Figure 4C); and 5) PFD only synergizes with 17-AAG to inhibit the K-Ras dependent growth *in vitro* (Figure 4D and Supporting information, Figure S4). Phosphorylated Hsp90 in the complex, on the other hand, may be an executor to maintain MT K-Ras protein in a proper conformation thereby preventing it from proteasome-dependent degradation (Figure 5B). Although Hsp90 can protect many onco-proteins from degradation [30] such as Bcr-Able [54], Raf-1 [55] and mutated B-Raf [35], its specific MT K-Ras protective effect may depends on p38γ. This is because the p38γ inhibitor PFD both blocks Hsp90 phosphorylation and down-regulates MT K-Ras expression (Figure 4B/6C/D). Therefore, three proteins in this ternary-complex are interdependent with a common signaling output to specifically sustain MT K-Ras protein expression and to selectively promote the K-Ras dependent growth and progression (Figure 6E).

K-Ras can be targeted by specific antisense oligos [56] or miRNA [57] but whether these approaches can specifically target MT K-Ras remains unknown. Our results reported here may indicate a novel therapeutic strategy against K-Ras-dependent malignancies, as targeting either p38γ or Hsp90 decreases MT, but not WT, K-Ras expression and effectively inhibit K-Ras MT colon cancer growth (Figure 1-6). However, we should be cautious about the specificity and efficacy of targeting Hsp90 alone, as Hsp90 can also act on the MT tumor suppressor p53 [58] and Hsp90 inhibition can induce Hsp70 thus decreasing the therapeutic efficacy [59]. But, our results of the combination strategy of the p38γ inhibitor PFD with the Hsp90 inhibitor 17-AAG to synergistically and selectively inhibit the K-Ras dependent growth (Fig. 4*d*) are very promising and may have a great application potential. While these inhibitors may have off-target effects, our results strongly indicate a cooperative role of p38γ and Hsp90 in maintaining MT K-Ras expression and consequently in driving the K-Ras dependent growth. Although the therapeutic advantages of PFD in combination with 17-AAG over either alone require further experimentations in mice to rule out potential synergistic toxicities *in vivo*, these results provide a proof of concept that a combined targeting the two steps in the complex can achieve more effective therapeutic effects against K-Ras dependent colon cancer. Previous studies showed that Hsp90 stabilizes the serine/threonine kinase STK33 leading to an increased sensitivity of K-Ras MT cancer cells to Hsp90 inhibitors but whether this kinase has any effect on the Ras oncoprotein or Hsp90 has not been demonstrated [60]. Moreover, while several Hsp90 inhibitors are currently in clinical trials against several types of human malignancies, their poor selectivity and severe toxicities have become a main concern [12]. Addition of the p38γ inhibitor PFD to the therapeutic regime with an Hsp90 inhibitor may increase its selectivity and/or reduce its toxicities. Most importantly, PFD is non-toxic [51] and alone is more effective in inhibiting K-Ras MT than WT colon cancer growth *in vitro* and/or *in vivo* (Figure 4/6). Therefore, this FDA approved anti-fibroblastic drug may have immediate benefits in its new application against K-Ras MT colon cancer.

## Materials and methods

### Constructs, cell lines, antibodies, inhibitors, and other reagents

His-tagged p38γ and p38α as well as Flag-tagged p38γ expressing constructs were provided by Dr. Jiahuai Han [40]. Flag-tagged human Hsp90α was a gift from Dr. Len Neckers [41], whereas retrovirus LZRS-K-Ras and the MKK6-p38γ constructs have been described previously [17, 19, 20]. WT and MT (G12V) K-Ras in HA-tagged pcDNA3 vector were kindly provided by Dr. Carol Williams or purchased from Guthrie cDNA resource center. The pLenti6/Block-iT system (Block-it™ U6 RNAi entry vector Kit, Cat: K4944-00 and Block-it™ Lentiviral RNAi expression system, Cat: K4943-00, Invitrogen) was used to clone sequences for shRNAs. The target sequences for individual genes are as following; luciferase (shLuc): GTGCGTTGCTAGTACCAAC (shLuc); human p38γ: CTCATGAAACATGAGAAGCTA for shp38γ#1 and GAAGGAGATCATGAAGGTGAC for shp38γ#2 [20]. Human colon cancer cell lines CaCo2, HT29, Ls-174T were purchased from ATCC and normal human colon epithelial NCM460 cells were bought from INCELL Corporation. HCT116 and its MT K-Ras disrupted sublines (Hke3 and HK2-8) were provided by Dr. Shirasawa [42]. Hsp90 protein inhibitor 17-AAG (geldanamycin) was from Selleck Chemicals (Cat. Number: S1141), whereas p38γ inhibitor pirfenidone (PFD) was purchased from Sigma (Product Number: P2116). Both drugs were dissolved in DMSO, which were stored at -20°C until use. The mouse phospho-antibodies (p-p38, p-ERK, p-JNK) and phosphorylated MAPK substrate (p-S/TP, Cat #:2325) were purchased from Cell Signaling. Other antibodies were provided by Santa Cruz, including mouse anti-K-Ras (sc-30), rabbit an-Hsp90 (sc-7947), mouse anti-Flag (sc-807), mouse anti-GAPDH (sc-47724), mouse anti-HA (sc-7392), mouse anti-α-Actinin (sc-17829) and rabbit anti-p38α (sc-535). Anti-p38γ goat and rabbit antibodies were bought from R&D Systems. Serum and other cell culture materials were from Gibco and all other chemicals were from Sigma.

### Proteomic analysis

The SDS-PAGE gel containing the proteins of interest as indicated (Fig. 1B) was cut, which was used for in gel digestion, TiO_2_ enrichment and LC-MS/MS analysis. The procedures for in gel digestion, sample preparation, and data analysis were as previously described [22].

### Statistical analysis

Results from all experiments, unless specified, were analyzed by student's t test for a statistical significant difference.

## AUTHORS' CONTRIBUTIONS

X.Q. performed animal studies, colony formation and viability assays, in vitro and in vivo Hsp90 phosphorylation experiments, Hsp90 sequence analysis and generating mutated Hsp90 constructs and cloning them into pcDNA3 vector, conducted K-Ras rescued experiments, stable transfection and infection experiments, established Ras transformed MEF cell lines, and performed transient transfection for protein-protein interaction by IPs/WB, and protein stability experiments. C.X. collected pathological specimens, performed IHC staining and patient survival analysis. S.H., G.C., M. C., and S.P.M. designed and/or performed proteomic analysis, and S.H. also generated Flag-p38γ stably expressed 116 and Hke-3 cell lines and p38γ depleted 116 cells, performed associated IP/WB analysis, and WB in a panel of colon cancer cells. N.Y. participated in colon cancer xenograft studies. G.L. and L.D. formed IHC staining, scoring, and statistical analysis. A.S. contributed to patient survival data analysis and S.T. assisted in manuscript editing, whereas Z.B. participated in pathological analysis and data interpretation. S.W. supervised pathological studies and was responsible for designing clinical experiments and patient survival data analysis. G.C. was responsible for designing all other experiments, performed animal studies, supervised overall projects, provided funding supports, interpreted results, and wrote the manuscript.

## SUPPLEMENTARY FIGURES


