# Supplementary material for: Identification of a ternary protein-complex as a therapeutic target for K-Ras-dependent colon cancer
Source: Oncotarget. 2014 May 25;5(12):4269–82. doi: 10.18632/oncotarget.2001 (PMC4147322; doi:10.18632/oncotarget.2001)
Supplement: Supplementary file 1 [file oncotarget-05-4269-s001.pdf]

Identification of a ternary protein-complex as a therapeutic target for K-Ras-dependent colon cancer

Supporting Information

Supplemental methods

Transfection, infection, and siRNA knockdown

Transient transfection was performed by calcium phosphate in 293T cells and by lipofectamine 2000 in NCM460 and 116 cells according to the Manufacturers' manuals. Hsp90/S595A was generated by site-directed mutagenesis using PCR-based techniques, which was further confirmed by DNA sequencing and cloned into pCDNA3 vector. Primers for Hsp90/S595A are: forward, 5'-ccg att ggt gac agc tcc atg ctg ta and reversed, 5'-caa tac agc atg gag ctg tca cca at. To stably express Hsp90/S495A in colon cancer cells, the mutant construct was transfected with calcium phosphatase and transfected cells were then selected with G418. To re-express K-Ras, Hke3 cells were infected with LZRS-K-Ras (G12V) or a LZRS vector, which were then selected with antibiotic as previously described [1]. To deplete p38γ protein, cells were infected with lentivirus expressing p38γ shRNA or control shLuc and then selected with antibiotic as described [2]. For retroviral infection, the construct was first transfected into package cells and supernatants were collected 48-72 later for infecting target cells, followed by an antibiotic selection [2].

Cell proliferation, cell viability, soft-agar assay, colony formation, and animal studies

Cells in 96 well-plate were incubated with different reagents as indicated for 24 hr and cell proliferation was performed according to the CyQuant NF kit manual (Cat: C35006) as previously described [3]. For colony formation in human colon cancer cells, 200 cells were plated in duplicate in a 6-well plate, and incubated with 17-AAG and/or PFD for about 2 weeks. The colonies formed were stained and counted as described [3]. For cell viability assay, cells were plated at the same density,

treated with 17-AAG or DMSO for 24 h, and viable cells were counted after staining with trypan blue [4]. Animal studies were conducted in accordance to the approved protocol by the Medical College of Wisconsin Institutional Animal Care and Use Committee. Because Ls-174T cells injected in PBS failed to form a tumor in nude mice (data not shown), we had to inject these cells in Matrigel as previously reported [5]. Briefly, indicated cells were s.c. injected into male Balb/c nude mice (Charles River) [2, 4] and therapy with 17-AAG or PFD (or DMSO) was initiated when tumor becomes palpable. Tumor-growth was measured using with a caliper as previously described [2], and dissected tumors were further photographed and weighed by the end of experiments.

Immunohistochemistry (IHC) and clinical patient survival data analysis

These studies were conducted in accordance to the approved protocol by IRB (Institutional Review Board). The IHC studies were performed using a specific antibody against p38γ and results were scored by two independent observers including a board-certified pathologist using criteria as previously described [2, 3]. Kaplan-Meier survival curves were generated for patients with tumors expressing low or high levels of p38γ protein expression using scores as previously defined [6]. Cancer-specific survival was calculated as the time interval from diagnosis to death due to the cancer. Patients who were alive with no evidence of the disease and those who died from inter-current disease were censored [7].

Kinase assays, immunoprecipitation, and immunoblotting

Kinase assays were performed as previously described [8-10]. For immunoprecipitation (IP), cells were harvested and lysed in RIPA buffer containing several phosphatase and protease inhibitors [2, 11]. To assess the complex formation in xenografts, tumor tissues were homogenized in RIPA buffer and lysates were prepared for IP/WB analysis as previously described [2]. The lysates were then pre-

cleared with protein A or G agarose beads, followed by incubating with the precipitating antibody overnight. Thereafter, the immune complexes were washed with RIPA buffer four times and the pellets were re-suspended in 1 X loading buffer, followed by boiling at 100°C for 5 min. Protein samples from total cell lysates (input) were resolved by SDS-PAGE, which were then transferred to a nitrocellulose membrane. The filter was blocked in 5% non-fat milk for 45 min and blotted with the appropriate antibody for 1 h at room temperature. The rest procedures were the same as previously described [2, 11].

Supplemental References

1. Tang J, Qi X, Mercola D, Han J, Chen G. Essential role of p38γ in K-Ras transformation independent of phosphorylation. J Biol Chem 2005; 280:23910-23917.
2. Hou SW, Zhi H, Pohl N, Loesch M, Qi X, Li R, Basir Z, Chen G. PTPH1 dephosphorylates and cooperates with p38γ MAPK to increases Ras oncogenesis through PDZ-mediated interaction. Cancer Res 2010; 70:2901-2910.
3. Zhi H, Hou S, Li R, Basir Z, Xiang Q, Szabo A, Chen G. PTPH1 cooperates with vitamin D receptor to stimulate breast cancer growth through their mutual stabilization. Oncogene 2011; 30:1706-1715
4. Qi X, Borowicz S, Pramanik R, Schultz RM, Han J, Chen G. Estrogen receptor inhibits c-Jun-dependent stress-induced cell death by binding and modifying c-Jun activity in human breast cancer cells. J Biol Chem 2004; 279:6769-6777.
5. Marin JJG, Macias RIR, Criado JJ, Bueno A, Monte MJ, Serrano MA. DNA interation and cytostatic activity of the new liver organotropic complex of cisplatin with glycocholic acid:
Bamet-R2. Int J Cancer 1998; 78:346-352.
6. Zhi H, Yang XJ, Kuhnmuench J, Berg T, Thill TBR, Yang H, See WA, Becker CG, Williams CL, Li R. SmgGDS is up-regulated in prostate carcinoma and promotes tumour phenotypes in prostate cancer cells. J Pathol 2009; 217:389-397.
7. Bleeker WA, Hayes VM, Karrenbeld A, Hosfstra RMW, Hermans J, Buys CCMea. **I**mpact of KRAS and TP53 mutations on survival in patients with left- and right sided dukes' C colon cancer. Am J Gastroenterol 2000; 95:2953-2957.
8. Chen G, Hitomi M, Han J, Stacey DW. The p38 pathway provides negative feedback to Ras proliferative signaling. J Biol Chem 2000; 275:38973-38980.
9. Hou S, Padmanaban S, Qi X, Leep A, Mirza S, Chen G. p38γ MAPK signals through phosphorylating its phosphatase PTPH1 in regulating Ras oncogenesis and stress response. J Biol Chem 2012; 287:27895-27905.
10. Qi X, Zhi H, Lepp A, Wang P, Huang J, Basir Z, Chitambar CR, Myers CR, Chen G. p38□ mitogen-activated protein kinase (MAPK) confers breast cancer hormone sensitivity by switching estrogen receptor (ER) signaling from classical to nonclassical pathway via stimulating ER phosphorylation and c-Jun transcription. J Biol Chem 2012; 287:14681-14691.
11. Qi X, Hou S, Lepp A, Li R, Basir Z, Lou Z, Chen G. Phosphorylation and stabilization of topoisomerase IIγ by p38γ MAPK sensitize breast cancer cells to its poisons. J Biol Chem 2011; 286:35883-35890.

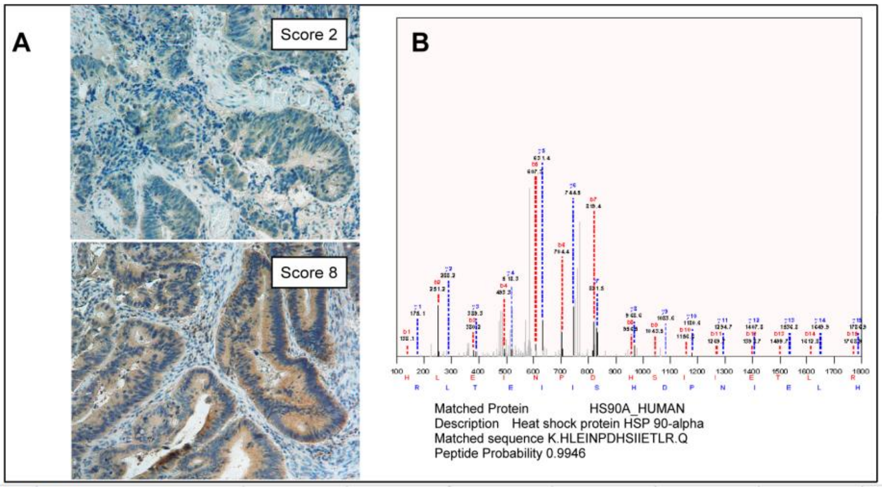

C

111210_1	116/sample 1								
Rank	Name	Accession	Descriptior	Protein Pr	Score	Peptide Cc	Run Count	Scan Cour	Total Xcorr
1	HS90A_HU	P07900	Heat shocl	1		3		5	19.6354
2	K2C1_HUI	P04264	Keratin, ty	1		3		4	15.7668
3	HS90B_HU	P08238	Heat shocl	0.999984		2		4	16.7645
4	K1C9_HUI	P35527	Keratin, ty	1		2		3	15.2649

Figure S1: p38γ expression is increased in primary CRC tissues and p38γ binds Hsp90 in K-Ras

MT colon cancer cells. (A) Representative IHC images of p38γ expression in colon cancer tissues. (B, C) Results in B represent MS/MS spectra of heat shock protein 90α peptide HLEINPDHSIIETLR from the number 1 of **Figure 1B** with representative peak matching for b and y ions (the SEQUEST search data are given C).

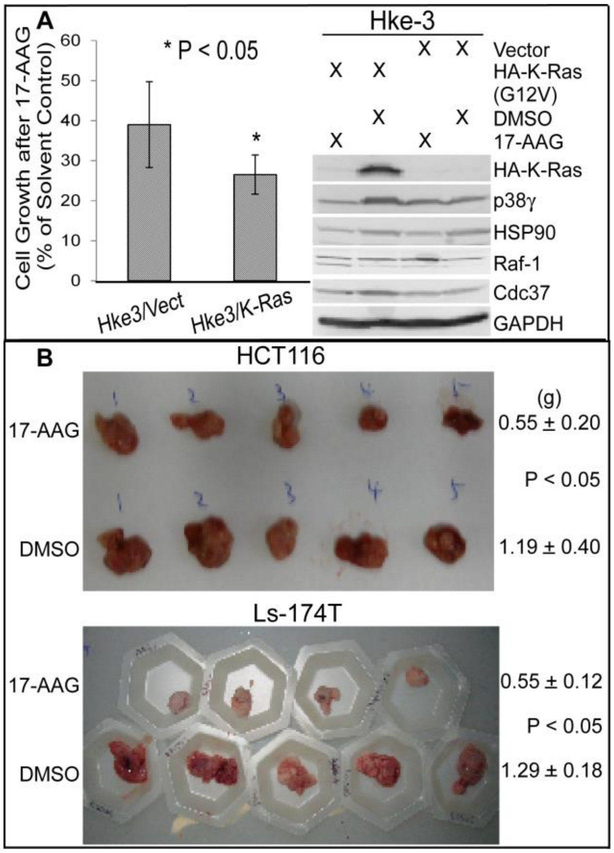

Figure S2: MT-K-Ras re-expression in Hke3 cells both increases p38γ expression and confers the sensitivity to 17-AAG-induced growth inhibition, and 17-AAG inhibits K-Ras MT colon cancer xenograft growth. **(**A**)** Hke3 cells were re-expressed with MT-K-Ras (G12V) by retroviral infection and resultant cells were incubated with 17-AAG (0.5 μM for 24 h) for growth inhibition (left) and protein expression (right). Cell growth were assessed using the CyQuant NF cell proliferation assay [3] (Left, mean ± SD, * P < 0.05 vs. Vector cells). (B) Tumors were removed from mice at the end of therapy with 17-AAG or DMSO, and were photographed and weighted (mean ± SD).

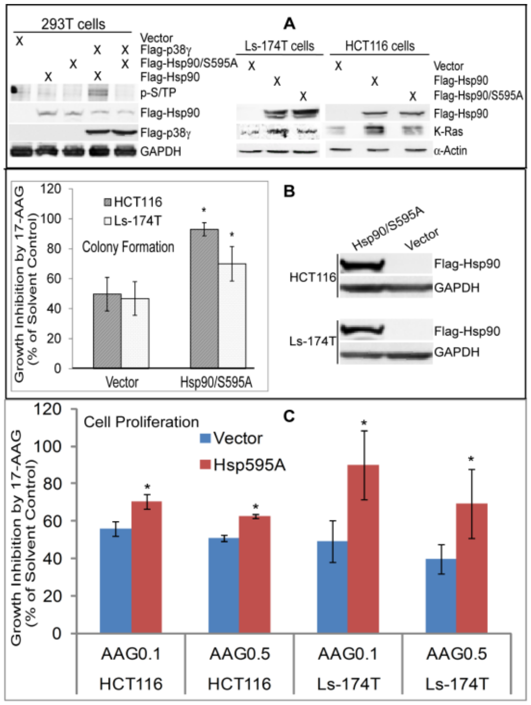

Figure S3: p38γ phosphorylates Hsp90 but not Hsp90/S595A, and Hsp90/S595A stable expression decreases K-Ras expression and suppresses the K-Ras-dependent growth *in vitro*. (A-C) Cells (293T) were transiently transfected with indicated constructs for 48 h and analyzed for protein expression/phosphorylation by WB (left). Flag-tagged WT Hsp90 and MT Hsp90/S595A were stably expressed in K-Ras MT HCT116 and Ls-174T colon cancer cells by antibiotic selection. Resultant cells were analyzed for protein expression (A, right and B, right) and for 17-AAG-induced growth inhibition by colony formation (B, left) and by the CyQuant NF cell proliferation assay (C) [3] (±SD, n = 3, * vs. vector transfected cells for B and C).

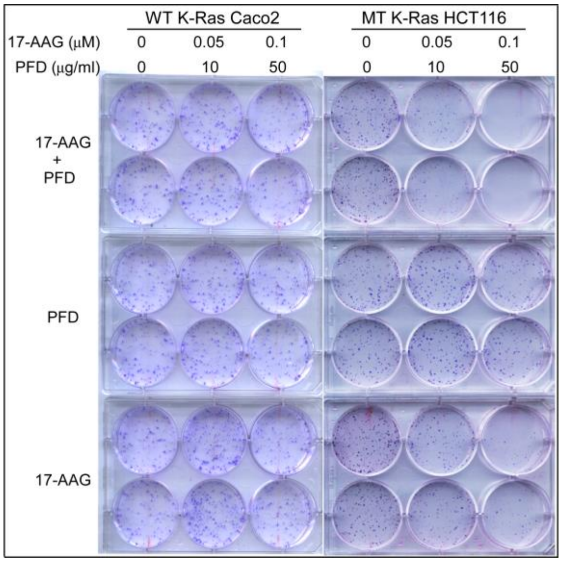

Figure S4: PFD synergizes with 17-AAG to inhibit the colony formation in K-Ras MT HCT116 but not in K-Ras WT Caco2 cells. Indicated cells were plated in 6-well plate in duplicate in the presence of PFD and/or 17-AAG (or solvent control) for about 2 weeks. Colony formed were fixed and stained, and plates from one representative experiment of three experiments were shown.

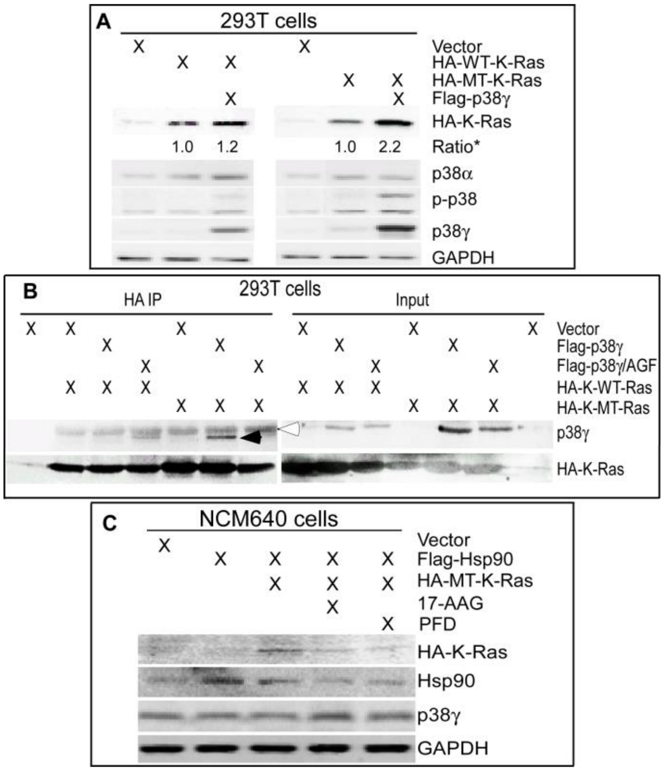

Figure S5: p38γ increases co-expressed MT, but not WT, K-Ras protein expression through a complex formation and treatment with 17-AAG or PFD decreases the exogenous MT K-Ras expression. (A**)** Cells were transiently transfected with indicated constructs and analyzed for protein expression by WB. The band intensity was measured with the NIH software and expressed as fold increase by p38γ co-transfection after normalized with GAPDH. (B) Cells were transfected with indicated constructs and HA immune-precipitates were analyzed for bound Flag-p38γ vs. Flag- p38γ/AGF by WB with a portion of whole cell lysates as input control (the open arrow head, IgG; the closed arrow head, Flag-p38γ). (C) NCM640 cells were transiently expressed with indicated constructs for 48 h with and without the last 24 h treatment with either 17-AAG or PFD. Cells were analyzed for protein expression by WB.
